# Plant-based dietary practices in Canada: examining definitions, prevalence and correlates of animal source food exclusions using nationally representative data from the 2015 Canadian Community Health Survey–Nutrition

**DOI:** 10.1017/S1368980020003444

**Published:** 2021-04

**Authors:** Mirjana Valdes, Annalijn Conklin, Gerry Veenstra, Jennifer L Black

**Affiliations:** 1 Food Nutrition and Health, Faculty of Land and Food Systems, University of British Columbia, 2205 East Mall, Vancouver, BC V6T 1Z4, Canada; 2 Faculty of Pharmaceutical Sciences, University of British Columbia, Vancouver, BC V6T 1Z3, Canada; 3 Department of Sociology, Faculty of Arts, University of British Columbia, Vancouver, BC V6T 1Z1, Canada

**Keywords:** Vegetarian, Plant-based diet, Canada, Dietary pattern, National health survey

## Abstract

**Objective:**

While plant-based dietary practices (PBDPs) have been recommended to improve both population health and environmental sustainability outcomes, no nationally representative Canadian studies have described the prevalence or correlates of excluding animal source foods. The current study therefore: (1) created operationalised definitions of PBDPs based on animal source food exclusions to estimate the prevalence of Canadians who adhere to PBDPs and (2) examined key correlates of PBDPs.

**Design:**

Population representative, cross-sectional data were from the 2015 Canadian Community Health Survey–Nutrition. Respondents’ PBDPs were categorised as: (1) vegan (excluded red meat, poultry, fish, eggs and dairy); (2) vegetarian (excluded red meat, poultry and fish); (3) pescatarian (excluded red meat and poultry) and (4) red meat excluder (excluded red meat). Descriptive statistics and multivariable regression analyses were used to examine the prevalence and correlates of these PBDP categories.

**Setting:**

All ten provinces in Canada.

**Participants:**

Canadians aged 2 years and above (*n* 20 477).

**Results:**

In 2015, approximately 5 % of Canadians reported adhering to any PBDP (all categories combined) with the majority (2·8 %) categorised as a red meat excluder, 1·3 % as vegetarian, 0·7 % as pescatarian and 0·3 % as vegan. South Asian cultural identity (OR 19·70 (95 % CI 9·53, 40·69)) and higher educational attainment (OR 1·97 (95 % CI 1·02, 3·80)) were significantly associated with reporting a vegetarian/vegan PBDP.

**Conclusions:**

Despite growing public discourse around PBDPs, only 5 % of Canadians reported PBDPs in 2015. Understanding the social and cultural factors that influence PBDPs is valuable for informing future strategies to promote environmentally sustainable dietary practices.

Plant-based dietary practices (PBDPs) have been widely recommended to improve population health and environmental sustainability outcomes^(1–3)^. For example, a vegetarian dietary pattern is recommended as a healthy dietary pattern in the United States Department of Agriculture’s 2015–2020 Dietary Guidelines for Americans^(4)^. The Academy of Nutrition and Dietetics has further stated that well-planned vegetarian diets (including vegan diets) can be nutritionally adequate, healthful and may contribute to the prevention and treatment of some diseases, and that plant-based diets use fewer natural resources and are less damaging to the environment^(1)^.

Proponents of PBDPs now draw on a growing literature to call attention to the potential benefits of dietary patterns rich in plant-based foods. For example, lacto-ovo-vegetarianism and veganism have been associated with lower odds of hypertension compared with non-vegetarians, in cohort studies of 7th Day Adventists^(5)^. Vegetarianism has further been associated with lower incidence of ischemic heart disease, as reported by a 2017 meta-analysis that included ten prospective cohort studies^(6)^. Plant-based diets are also associated with a lower prevalence of type 2 diabetes and have been recommended by Diabetes Canada for medical nutrition therapy in type 2 diabetes management^(7)^. Recently, the 2019 EAT-Lancet Commission further proposed a ‘universal healthy reference diet’ which recommends a diet higher in diverse plant-based foods, with limited amounts of seafood and poultry, and low to no red and processed meat to improve both human health and sustainability of the planet^(3)^. Furthermore, one systematic review which included sixty-three studies assessing shifts to more sustainable dietary patterns suggested that moving towards veganism and vegetarianism from current Western diets would contribute to the highest reductions in greenhouse gas emissions and land use compared with other dietary patterns^(2)^.

In Canada, discourse surrounding PBDPs has escalated following the 2019 release of the updated Food Guide. The new food guidance recommends a shift towards dietary patterns emphasising plant-based foods, especially plant-based sources of protein, not only to encourage reduced intakes of processed meat and foods high in saturated fat but also to promote conservation of soil, water and air^(8)^. However, little empirical attention to date has documented the diverse definitions used to assess PBDPs in Canada, or the extent to which Canadians actually follow different types of PBDPs.

Using provincially representative data on adults aged 19–84 years in 2005, Bedford and Barr estimated that 5·8 % of British Columbians self-identified as vegetarian, but most respondents did not report adhering to a strictly meatless diet^(9)^. Of the self-identified vegetarians, 22·4 % reported at least occasional consumption of red meat and 57·6 % consumed poultry^(9)^. Bedford and Barr found that self-identified vegetarian respondents in British Columbia were more likely to be women, younger and single and report low-income status compared with non-vegetarians^(9)^. More recently, findings from a 2019 convenience sample of 2566 youth and young adults (16–30 years old) in five major cities showed that 13·6 % reported some type of PBDP, with 6·6 % reporting vegetarianism, 4·5 % reporting pescatarianism and 2·5 % reporting veganism^(10)^. That survey also found that respondents who identified as South Asian only were more likely to be vegetarian than those who identified as White only, Black only, Chinese only, Aboriginal or mixed/other ethnicity, and that respondents identifying as female sex at birth were more likely to be pescatarian compared with self-identified males^(10)^. The true prevalence of PBDPs in Canada is currently unknown due to limited geographic coverage and selection bias of current studies.

The objectives of the current study were therefore to carefully operationalise definitions of PBDPs based on animal source food exclusions to estimate the Canadian prevalence of PBDPs in 2015 and to identify salient socio-demographic correlates of PBDPs in Canada.

## Methods

### Data source

Data were drawn from the ‘Health Component’ questionnaire from the 2015 Canadian Community Health Survey–Nutrition (CCHS). The CCHS was a population representative, cross-sectional survey focused on characterising dietary patterns, supplement intake and socio-demographic and health characteristics of Canadians aged 2 years and above from the ten provinces of Canada^(11)^. Individuals who were full-time members of the Canadian Forces, lived in the Territories, on reserves, in some remote areas or in institutions (e.g., prisons or care facilities) were excluded. A multi-stage cluster sampling design was used to ensure that the survey was nationally and provincially representative of the population in terms of age, sex, geography and socio-economic status with a response rate of 61·6 %^(11)^.

The CCHS is an optimal dataset to explore PBDPs as it is the first nationally representative survey in Canada to include a question about complete dietary exclusion of animal source food products. This question was specifically developed for the 2015 survey to examine different types of vegetarianism and only ten participants did not answer the dietary exclusion question^(12)^. This recently added question asked the following: ‘Do you completely exclude any of the following foods from your diet? By “completely exclude” we mean you never eat it on its own or as part of a prepared dish (Meat (beef, pork, lamb, etc.); Poultry (chicken, turkey, duck, etc.); Fish and shellfish; Eggs; Dairy products (milk, cheese, etc.); Gluten sources (wheat, barley, rye, etc.); None)’; respondents had the option to choose as many of these categories that they ‘completely excluded’^(11)^. The analytical sample included participants who answered the dietary exclusion question (*n* 20 477). As the current study involved secondary data analysis of anonymised data collected under the authority of the Statistics Act^(13)^, approval to conduct this research was obtained from the Statistics Canada Research Data Centre Program^(14)^.

#### Plant-based dietary practice outcome variables

The definitions of PBDPs were operationalised from the responses to the dietary exclusion question (see Table 1). To ensure that no exclusion combination was overlooked, a user-generated command in Stata ‘groups.ado’ was run^(15)^. This command listed all frequencies for every possible iteration of the dietary exclusion combinations. Each combination of exclusions was reviewed and manually coded into one of four PBDP categories based on a priori definitions informed by how each PBDP category had been defined in the literature. Respondents that reported excluding red meat, poultry, fish and shellfish, eggs and dairy were coded as ‘Vegans’. ‘Vegetarians’ were coded as those that reported excluding at least red meat, poultry, fish and shellfish with no restrictions on eggs, or dairy. ‘Pescatarians’ were defined as those who reported excluding red meat and poultry. ‘Red Meat Excluders’ were defined as those who reported excluding red meat. All other dietary exclusion combinations including exclusions that did not align with the four PBDPs and those who did not report any exclusions were categorised as ‘Non-PBDP’. The assigned categories were mutually exclusive.

**Table 1:** Operationalised definitions of plant-based dietary practices from the Canadian Community Health Survey–Nutrition 2015

PBDP category	Defined by exclusion of*:				
	Red meat	Poultry	Fish and shellfish	Eggs	Dairy products
Vegan	X	X	X	X	X
Vegetarian	X	X	X		
Pescatarian	X	X			
Red meat excluder	X				
Non-PBDP					

PBDP = plant-based dietary practice.

* The full survey question asked: ‘Do you completely exclude any of the following foods from your diet? By “completely exclude” we mean you never eat it on its own or as part of a prepared dish.’; If an exclusion (out of the categories of meat, fish, poultry, eggs, dairy products and gluten) is not listed, the respondent may or may not have excluded them in their diet, for example, a ‘Vegetarian’ respondent excluded meat, fish and poultry but may or may not have excluded eggs or dairy products.

#### Independent variables

Key socio-demographic correlates available in the CCHS dataset included: province of residence Atlantic (including Nova Scotia, New Brunswick, Newfoundland and Prince Edward Island), Quebec, Ontario, Prairie (including Alberta, Manitoba and Saskatchewan) and British Columbia), urban/rural residential location, gender (women/men), age (in years), marital status (partnered (married/living as married) *v*. non-partnered (single, widowed and divorced/separated)), immigration status, highest education attained in the household (high school equivalent or below; certificate or diploma below bachelor’s level; bachelor’s degree or higher), employment status in previous week (employed/unemployed), food insecurity (food secure/food insecure based on the 18-item US Household Food Security Survey Model questionnaire)^(11)^, total household income in quintiles (lowest quintile = 1 to highest quintile = 5), supplement use in previous month (yes/no), participation in 150 min per week of ‘moderate or vigorous’ physical activity (yes/no), chronic disease status (high blood pressure, diabetes, heart disease and/or cancer), smoker/non-smoker and measured BMI (kg/m^2^).

The construct of self-identified cultural identity was collected by asking respondents to choose as many ‘racial or cultural groups’ they belonged to from a predefined list: White, South Asian, Chinese, Black, Filipino, Latin American, Arab, Southeast Asian, West Asian, Korean, Japanese and Other^(16)^. Self-identified cultural identity was collapsed into a three-category variable for analysis (White only, South Asian only and Other). The Other category included respondents that identified as non-White only, non-South Asian only or persons who reported more than one cultural identity.

### Statistical analysis

To estimate the prevalence of PBDPs in Canada, survey-weighted frequency tables were constructed using the operationalised definitions of PBDPs (*n* 20 477). To explore bivariate associations between the independent variables of interest and PBDP categories, data were examined in weighted frequency distribution tables and analysed using Rao–Scott *χ* ^2^ tests^(17)^. For continuous variables, a simple linear regression model was used with PBDP category as the independent variable, and the continuous variable (age or BMI) served as the dependent variable. A *P*-value of < 0·05 was defined as statistically significant, with a Bonferroni correction applied to account for multiple comparisons for the analyses on continuous variables. To meet Statistics Canada reporting requirements for minimum cell size, ‘Vegan’ and ‘Vegetarian’ categories were combined in Tables 3 and 4. For this same reason, other categorical independent variables were also collapsed into fewer levels. Sensitivity analyses were conducted to make sure the collapse of categories did not substantially affect the magnitude and direction of associations between PBDPs and independent variables. Demographic characteristics were described for the entire sample (aged 2 years and above) or as appropriate (e.g., smoking was collected only for respondents aged 12 years and over). Missing data were deleted on a case-wise basis and comprised < 1 % of the total sample size for each characteristic in Table 3, except for BMI for which 7 % of the samples were missing data.

**Table 2:** Weighted prevalence of plant-based dietary practices among Canadians aged ≥2 years from the Canadian Community Health Survey–Nutrition 2015 (*n* 20 477)

Theoretical PBDP category	%	se	Defined as those who excluded:
Vegan	0·28	0·08	Red meat, fish, poultry, eggs, dairy
Vegetarian	1·29	0·16	Red meat, fish, poultry
Pescatarian	0·65	0·13	Red meat, poultry
Red meat excluder	2·81	0·23	Red meat
Non-PBDP	94·97	0·32	
Total	100		

PBDP = plant-based dietary practice.

**Table 3:** Weighted prevalence of plant-based dietary practices by independent variables of interest for Canadians ≥2 years old from the Canadian Community Health Survey–Nutrition 2015†,‡

Characteristics	PBDP categories								
	Vegetarians/vegans		Pescatarians		Red meat excluders		Non-PBDP		
	%	se	%	se	%	se	se	%	*P*
Total (*n* 20 477)	1·57	0·18	0·65	0·13	2·81	0·23	94·97	0·32	
Region of Canada§									0·009
Atlantic provinces	1·22	0·29	0·56	0·21	1·86	0·37	96·36	0·51	
Quebec	0·44	0·13	0·66	0·38	2·34	0·49	96·56	0·63	
Ontario	2·17	0·38	0·77	0·22	3·37	0·45	93·69	0·63	
Prairie provinces	1·36	0·29	0·35	0·10	2·63	0·34	95·66	0·49	
British Columbia	2·25	0·63	0·76	0·21	2·73	0·48	94·27	0·79	
Urban/rural residence									0·027
Urban	1·73	0·22	0·72	0·15	2·99	0·25	94·56	0·36	
Rural	0·77	0·22	0·32	0·19	2·00	0·53	96·92	0·67	
Gender									0·038
Women	1·89	0·29	0·69	0·14	3·35	0·34	94·07	0·46	
Men	1·23	0·20	0·62	0·22	2·26	0·26	95·89	0·38	
Marital status‖									0·078
Non-partnered	1·14	0·23	1·05	0·31	2·53	0·40	95·27	0·56	
Partnered	1·88	0·29	0·55	0·15	2·96	0·33	94·61	0·45	
Immigrated to Canada¶									<0·001
Yes	3·94	0·59	1·00	0·34	4·76	0·66	90·29	0·92	
No	0·82	0·13	0·54	0·13	2·21	0·22	96·43	0·29	
Cultural identity†,†									<0·001
White	0·67	0·13	0·53	0·13	1·72	0·19	97·08	0·26	
South Asian	14·41	2·23	0·65	0·38	13·38	2·08	71·56	3·03	
Other	1·50	0·42	1·11	0·40	4·14	0·63	93·24	0·82	
Education‡,‡									<0·001
High school equivalent	1·04	0·24	0·27	0·09	2·14	0·34	96·55	0·43	
Certificate/diploma below bachelor’s degree	0·92	0·18	0·68	0·26	2·12	0·31	96·28	0·45	
Bachelor’s degree or higher	2·48	0·37	0·84	0·20	3·85	0·42	92·83	0·61	
Employment status§,§									0·335
Unemployed	1·60	0·30	0·64	0·17	3·48	0·48	94·27	0·57	
Employed	1·69	0·28	0·79	0·22	2·52	0·29	95	0·47	
Household food insecurity‖,‖									0·846
Food secure	1·60	0·19	0·67	0·14	2·81	0·23	94·93	0·33	
Food insecure	1·22	0·44	0·51	0·24	2·92	0·60	95·35	0·85	
Household income¶,¶									0·123
Quintile 1	1·98	0·40	1·05	0·43	3·96	0·60	93·01	0·79	
Quintile 2	1·99	0·45	0·34	0·13	2·85	0·44	94·82	0·72	
Quintile 3	1·60	0·44	0·84	0·37	2·74	0·45	94·82	0·70	
Quintile 4	1·16	0·27	0·57	0·23	2·34	0·52	95·94	0·67	
Quintile 5	1·08	0·35	0·46	0·14	2·16	0·43	96·31	0·55	
Supplement intake†,†,†									0·012
Yes	2·16	0·29	0·82	0·20	3·03	0·33	94·00	0·48	
No	1·08	0·20	0·51	0·17	2·63	0·29	95·78	0·41	
Participated in 150 min of physical activity/week‡,‡,‡									0·303
Yes	1·14	0·27	0·81	0·24	2·71	0·38	95·34	0·49	
No	1·92	0·30	0·70	0·21	2·88	0·35	94·51	0·54	
Has at least one chronic disease§,§,§									0·261
Yes	1·80	0·49	0·38	0·10	3·33	0·57	94·50	0·70	
No	1·48	0·21	0·82	0·21	2·66	0·29	95·03	0·42	
Smoker‖,‖,‖									<0·001
Yes	0·32	0·17	0·42	0·20	1·28	0·32	97·98	0·41	
No	1·87	0·25	0·79	0·17	3·14	0·29	94·20	0·41	
Age¶,¶,¶									0·315
Mean	37·87		41·03		41·32		41·28		
se	1·79		2·25		1·35		0·13		
BMI†,†,†,†									<0·001
Mean	25·37*		24·93*,**		26·58*,**		27·18**		
se	0·56		0·87		0·49		0·10		

PBDP, plant-based dietary practice.

† *P*-values for categorical variables generated from *χ* ^2^ test with Rao–Scott correction.

‡ *P*-values for continuous variables (age and BMI) generated from *F*-test for the simple linear regression model; if the model was significant, a Bonferroni correction was applied to check which marginal means were significantly different from each other.

§ Atlantic provinces include Nova Scotia, New Brunswick, Newfoundland and Prince Edward Island; Prairie provinces include Alberta, Manitoba and Saskatchewan.

‖ Non-partnered includes widowed and separated; partnered includes married and common law; valid *n* 14 969.

¶ Valid *n* 20 446.

†,† ‘Other’ includes respondents that identified as non-White only, non-South Asian only or who reported more than one cultural identity; valid *n* 19 496.

‡,‡ This corresponds to the highest education attained in the household; levels include up to high school equivalent, certificate or diploma below bachelor’s level and bachelor’s degree or higher; valid *n* 20 437.

§,§ Refers to employment status in the last week; This information was collected for respondents aged 15–75 years; valid *n* 13 736.

‖,‖ Valid *n* 20 365.

¶,¶ Total household income before taxes.

†,†,† Refers to supplement intake in the past month; valid *n* 20 466.

‡,‡,‡ Data collected for those aged 18 years+ only; valid *n* 14 221.

§,§,§ Has at least one chronic disease out of high blood pressure, diabetes, heart disease or cancer; data collected for those aged 19 years+ only; valid *n* 13 851.

‖,‖,‖ Data collected for those aged 12 years+ only; valid *n* 16 646.

¶,¶,¶ Age measured in years; age also measured as a categorical variable according to Dietary Reference Intake cutoffs was also not significant (data not shown due to vetting restrictions regarding low cell counts by Statistics Canada).

†,†,†,† Measured BMI was used (70 % of respondents); when measured BMI was unavailable, reported BMI was used; means sharing a symbol (*,**) are not significantly different; refers to adult BMI and only applicable for non-pregnant respondents aged 18 years+; valid *n* 12 574.

**Table 4:** Adjusted OR of reporting red meat exclusion/pescatarianism or vegetarianism/veganism relative to no plant-based dietary practice among Canadians (16+) from the Canadian Community Health Survey–Nutrition 2015 (*n* 14 296)†

	Odds of red meat exclusion/pescatarianism		Odds of vegetarianism/veganism	
	OR	95 % CI	OR	95 % CI
Gender				
Men	Reference		Reference	
Women	1·50*	1·05, 2·12	1·75	0·98, 3·10
Age‡	1·01	1·00,1·01	1·00	0·98, 1·02
Urban/rural residence				
Rural	Reference		Reference	
Urban	1·10	0·59, 2·04	1·70	0·78, 3·70
Region of Canada§				
Ontario	Reference		Reference	
Atlantic provinces	0·89	0·52,1·50	1·89	0·84, 4·28
Quebec	0·96	0·56,1·67	0·47	0·19, 1·12
Prairie provinces	0·69	0·47, 1·01	0·80	0·42, 1·51
British Columbia	0·96	0·61,1·50	1·02	0·41, 2·55
Cultural identity‖				
White	Reference		Reference	
South Asian	7·98***	4·30,14·81	19·70***	9·53, 40·69
Other	1·96**	1·21, 3·17	1·67	0·80, 3·49
Immigrated to Canada				
No	Reference		Reference	
Yes	0·97	0·60, 1·58	1·59	0·87, 2·93
Education¶				
High school equivalent	Reference		Reference	
Certificate/diploma below bachelor’s degree	1·31	0·77, 2·25	0·83	0·38, 1·79
Bachelor’s degree or higher	2·03**	1·22, 3·39	1·97*	1·02, 3·80
Marital status††				
Non-partnered	Reference		Reference	
Partnered	0·88	0·61, 1·27	1·29	0·77, 2·14
Household income‡‡				
Quintile 1	Reference		Reference	
Quintile 2	0·61	0·36, 1·03	0·93	0·45, 1·94
Quintile 3	0·92	0·53, 1·60	1·32	0·56, 3·10
Quintile 4	0·69	0·39, 1·21	0·80	0·36, 1·79
Quintile 5	0·68	0·37, 1·28	0·93	0·39, 2·20

† Model includes sex, age, urban/rural residence, province of residence, self-identified racial/cultural grouping, immigration status, education, marital status and income. Cases with missing data were dropped (total valid *n* 14 296).

‡ Age measured in years.

§ Atlantic provinces include Nova Scotia, New Brunswick, Newfoundland and Prince Edward Island; Prairie provinces include Alberta, Manitoba and Saskatchewan.

‖ ‘Other’ includes respondents who identified as non-White only, non-South Asian only or who reported more than one cultural identity.

¶ This corresponds to the highest education attained in the household; levels include up to high school equivalent, certificate or diploma below bachelor’s level and bachelor’s degree or higher.

†† Non-partnered includes widowed and separated; partnered includes married and common law.

‡‡ Total household income before taxes * *P* < 0·05, **P < 0·01, ***P < 0·001.

A multinomial logistic regression model was run with the PBDP outcome variable collapsed into three groups: ‘Vegetarian/Vegans’, ‘Pescatarian/Meat-excluders’ and ‘Non-PBDP’ to investigate potential socio-demographic correlates associated with the odds of reporting total animal flesh exclusions (Vegetarian/Vegans) *v*. some meat exclusion (Pescatarian/Red Meat-Excluders) relative to those who did not exclude red meat from the diet. Socio-demographic variables were included in the model using a theory-driven method based on consistency of reported associations in the literature. Gender was included in the model because both measures of sex and gender have been shown to be consistently associated with plant-based eating, with females being more likely to report vegetarianism^(9,18–21)^. Several studies have also suggested an association between vegetarianism and younger age^(9,19,20,22–25)^. Geographic factors such as urban residence and Canadian province of residence were included in the model as these have been shown to be associated with plant-based eating^(22,24)^. Ethnicity and immigration status were included in the model as the prevalence of PBDPs has been shown to differ between countries^(18,23,26–29)^, and views on meat eating have also been shown to differ by culture^(30,31)^. Education and income, which encompass aspects of socio-economic status, and marital status were included in the model as these variables have been associated with vegetarian status in previous studies^(9,18,22,23,26)^. Marital status data were not obtained from respondents under the age of 16, so the multivariable model was restricted to respondents aged 16 years and above (*n* 14 296).

Owing to the complex sampling design used in the CCHS, survey sampling weights were applied to all analyses to account for unequal selection probabilities, and se were estimated using the 500 sets of replication weights provided by Statistics Canada^(12)^. All data analysis was conducted in the British Columbia Inter-University Research Data Centre using Stata version 13.

## Results

Table 2 describes the estimated prevalence of PBDPs in 2015, where the majority of Canadians (95 %) reported none of the PBDPs measured here. Among the 5 % of Canadians who did report adhering to a PBDP, the most common category was red meat exclusion (2·8 %), followed by vegetarianism (1·3 %), pescatarianism (0·7 %) and veganism (0·3 %). Online supplementary material, Supplemental Table 1 further provides detailed prevalence estimates for several other reported exclusion categories, beyond the PBDP categories under investigation here.

Table 3 describes how the prevalence of vegetarians and vegans combined, pescatarians, red meat excluders and not reporting any PBDPs differed across key socio-demographic factors including geographic region and urban/rural location. British Columbia (2·3 %) and Ontario (2·2 %) were the provinces with the highest reported proportion of vegetarians/vegans, and Ontario was also the region with the greatest proportion of red meat excluders (3·4 %). Moreover, a higher proportion of respondents also reported following PBDPs in urban areas (1·7 % for vegetarian/vegans, 0·7 % for pescatarians and 3·0 % for red meat excluders) compared with rural areas (0·8 % for vegetarians/vegans, 0·3 % for pescatarians and 2·0 % for red meat excluders).

Socio-cultural factors also emerged as salient, with a higher proportion of women reporting vegetarianism/veganism (1·9 %), pescatarianism (0·7 %) and red meat exclusion (3·4 %) relative to men (1·2, 0·6 and 0·3 %, respectively). Moreover, a higher proportion of respondents living in households where a person had achieved higher levels of educational attainment (bachelor’s degree or higher) reported vegetarianism/veganism (2·5 %), pescatarianism (0·8 %) and red meat exclusion (3·9 %) relative to those living in households with lower highest educational attainment (high school completion or below). Among respondents who reported immigrating to Canada, there was a higher proportion of PBDPs reported (3·9 % vegetarian/vegan, 1·0 % pescatarian and 4·8 % meat excluder) than among respondents who did not immigrate to Canada (0·8, 0·5 and 2·2 %, respectively). Among respondents who self-identified as South Asian, there was a far higher proportion of vegetarians/vegans (14·4 %) and red meat excluders (13·4 %) than among respondents identifying as White (0·7 and 1·7 %, respectively).

PBDP adherence was also associated with some but not all health-related variables examined here. For example, vitamin and mineral supplement users reported higher proportion of vegetarian/vegans (2·2 %), pescatarians (0·8 %) and red meat excluders (3·0 %) compared with those who did not report taking supplements in the last month (1·1, 0·5 and 2·6 %, respectively). Respondents who smoked reported lower prevalence of PBDP adherence (0·3 % vegetarian, 0·4 % pescatarian and 1·3 % red meat excluder) relative to non-smokers (1·9, 0·8 and 3·1 %, respectively). The mean BMI of vegetarians was significantly lower (25·4 kg/m^2^ (se 0·56)) than that of those who did not report any PBDP (27·2 kg/m^2^ (se 0·10)). Marital status, employment status, food insecurity, income, physical activity and reporting a chronic disease were not significantly associated with reporting PBDPs. Further, neither age in years nor age groups were statistically significant correlates of PBDPs.

Results of the adjusted multinomial logistic regression model are presented in Table 4. After adjustment for covariates, women were still more likely than men to report red meat exclusion/pescatarianism (OR 1·50 (95 % CI 1·05, 2·12)) compared with reporting no PBDP but did not significantly differ in the odds of vegetarianism/veganism. Moreover, compared with respondents in households with low educational attainment in the household, respondents living in households with the highest educational attainment were also more likely to report red meat exclusion/pescatarianism (OR 2·03 (95 % CI 1·22, 3·39)) and vegetarianism/veganism (OR 1·97 (95 % CI 1·02, 3·80)) compared with reporting no PBDP.

Self-reported cultural/racial identity emerged as a strong correlate of PBDPs in adjusted models. For example, the odds of reporting red meat exclusion/pescatarianism compared with not reporting a PBDP (OR 1·96 (95 % CI 1·21, 3·17)) were higher among participants who were categorised culturally as Other relative to White. Further, those who self-identified as South Asian were nearly eight times more likely to report red meat exclusion/pescatarianism (OR 7·98 (95 % CI 4·30, 14·81)) and nearly twenty times more likely to report vegetarianism/veganism (OR 19·70 (95 % CI 9·53, 40·69)), rather than no PBDP adherence, relative to White-identifying respondents.

Consistent with associations that were not statistically significantly reported in unadjusted analyses in Table 3, age, income and marital status remained not statistically significantly associated with PBDPs in adjusted models. However, after adjustment, associations between region of Canada, urban/rural residence and immigration status with red meat exclusion/pescatarianism and vegetarianism/veganism were also attenuated and failed to reach statistical significance.

## Discussion

The current study is the first to our knowledge to explore the prevalence and correlates of animal source food exclusions in Canada using population representative data. The contribution to the literature from this nationally representative study is timely given the release of the 2019 version of Canada’s Food Guide which now emphasises the consumption of plant-based foods^(32)^. It also coincides with the release of the 2019 EAT-Lancet Commission report which urges people to consume fewer meat products for the health and sustainability of the planet^(3)^. As such, the insights from the current study add valuable empirical findings from the Canadian context to inform the knowledge base regarding national prevalence and key correlates of PBDPs using rigorously and systematically constructed measures of animal source food exclusions.

The current study found that in 2015, relatively few Canadians (< 5 % the population) reported any PBDPs measured, with red meat exclusion reported as the most common dietary exclusion practice considered here (2·8 %), while 1·3 % of the population reported a vegetarian-style dietary practice, and < 1 % reported a pescatarian (0·7 %) or vegan (0·3 %) dietary practice. These estimates are considerably lower than recent findings reported in a 2018 media report of a Canadian poll from Dalhousie University which stated that 7·1 % of sampled Canadians considered themselves as vegetarian and 2·3 % were vegan^(24)^. Current PBDP estimates from the CCHS dataset are also lower than another Dalhousie-based 2018 survey report of 1027 adults showing that 3·3 % of the sample identified as vegetarian, 1·2 % as pescatarian and 1·1 % as vegan^(33)^. The discrepancies between findings from the current CCHS data and the 2018 polls could indicate rising interest in PBDPs in Canada since the CCHS was collected in 2015. However, it is more likely that differences in study methodology and sample size could explain the discrepancies between studies since the 2018 estimates stem from consumer polling which may upwardly bias the estimates compared with CCHS’s multi-stage cluster sample, designed to be representative of age, sex, geographic region and socio-economic status^(11)^. The current CCHS-based findings also differ from the results from Vergeer *et al.* who reported that 14 % of Canadian youth and young adults follow a PBDP^(10)^. Differences from CCHS estimates could also be due to sampling differences compared with Vergeer’s study, which focused on Canadians aged 16–30 living in major cities *v*. CCHS data collected from respondents living in both urban and rural locations across the ten provinces of Canada^(10)^. Vergeer *et al.* also assessed PBDP status using the self-report vegetarian status and did not provide definitions of different PBDP practices to respondents during their survey^(10)^.

When comparing data from the current study to other international estimates of PBDPs using data sources with similar methodology to the CCHS (i.e., nationally representative surveys), it also appears that CCHS-based estimates were lower than the majority of the international studies with the exceptions of estimates from Italy and Ireland. For example, the prevalence of vegetarianism in Italy in 2004 was reported at 0·79 %^(26)^, while the prevalence in Ireland in 2007 was 0·9 %^(27)^, both below the 1·3 % estimated prevalence of vegetarianism reported here in Canada in 2015. Still, the Canadian prevalence of vegetarianism (1·3 %) was lower than the estimated prevalence of vegetarianism in Germany from 2014 (2·5 %)^(28)^, Finland from 1997, 2000 and 2002 (3·3 %)^(23)^, the USA from 2012 (4·0 %)^(18)^ and India from 2006 (27·4 % lacto and lacto-ovo vegetarians)^(29)^. While these findings could reflect true differences in vegetarianism worldwide, discrepancies could also be from differences in definitions and measures of PBDPs.

Most of the prevalence estimates with the exception of those in Ireland and Finland used survey questions to assess PBDP adherence^(18,23,26–29)^. Specifically, while the definitions used here based on CCHS’s dietary exclusion question inquired about self-reported combinations of animal source food exclusions, most other studies asked participants variations of the question ‘are you a vegetarian?’. Studies conducted in Italy and America^(18,26)^ did not appear to provide definitions regarding the terms ‘vegetarian’ and ‘vegan’ at the time the survey questions were administered. Similarly, in Finland where both self-identified vegetarian status and FFQ data were collected, there was no definition for ‘vegetarian’ provided when the self-identified vegetarian survey question was asked. Thus, it is possible that respondents in these studies may have different ideas about what constitutes vegetarianism and as such the validity of the survey question in measuring the construct of ‘vegetarianism’ may be compromised.

In Ireland and Finland, FFQ were used to determine PBDP status, the definitions of which may be more similar to the ones used in the present study as both FFQ and the CCHS survey questions probe and define vegetarianism based on whether a respondent completely excludes certain foods^(23,27)^. In addition, the study conducted in India asked how often a participant consumed certain food groups with the response choices ranging from ‘daily, weekly, occasionally or never’, similar to the CCHS definitions of PBDPs in that respondents who did not consume flesh-based foods were categorised into the spectrum of PBDPs ranging from vegan to semi-vegetarian^(29)^. Thus, by using CCHS data to create definitions, the current study is still able to capture the subtle differences in animal source food exclusion in each PBDP type that provide more specific definitions than those created using a self-report vegetarian status question, while also providing lower respondent burden than completing a full FFQ.

The current study also sheds light on several important correlates of PBDPs. The most salient variables that were significant after multivariable adjustment and robust to sensitivity analyses assessing alternate approaches to variable construction included cultural/racial identity, gender and educational attainment, in directions that were consistent with previous studies^(10,18,22,23,26,28,34)^. However, while cross-tabulations suggested that PBDPs were associated with other socio-demographic characteristics such as region of Canadian residence, urban/rural residence and immigration status, these associations were attenuated after statistical adjustment, suggesting that the relevance of these factors is by-products of their confounding with other potentially more salient determinants such as cultural identity that shapes taste, preference and dietary habits particularly regarding meat consumption^(30,31,35)^.

The current study found that women had higher odds of reporting meat-exclusionary diets (including pescatarianism) relative to men, but this association was not statistically significant in regard to the odds of reporting vegetarianism/veganism compared with no PBDP adherence. Similarly, Vergeer *et al.* found that females relative to males were more likely to report pescatarianism (OR 2·45 (95 % CI 1·57, 3·81)) but that sex was not a significant predictor in the models focused on vegans or vegetarians^(10)^. In contrast, Bedford and Barr found that compared with non-vegetarians, among those who reported vegetarianism there was a significantly higher percent of women than men^(9)^. Although Bedford and Barr’s data were collected only in British Columbia, Canada, they also reported that 75 % of the self-identified vegetarians in their study consumed fish, so it is possible there were a high number of pescatarians within the self-identified vegetarian group^(9)^.

While studies in Canada suggest meat-exclusionary diets are more common among women, it is interesting to note that the association between vegetarianism and feminine gender (often measured with a dichotomised variable) is reported consistently in other population-based studies about vegetarianism from South Asia, America, Germany, Finland and Italy^(18,22,23,26,28,34)^. Pirani and Fegitz argue that mainstream representations of veganism depict a largely female and highly gendered position on meat exclusion that embodies ‘normative femininity’^(36)^. Their arguments are also supported by evidence of the association of meat and masculinity reported across different cultures^(37)^. For instance, in a nutritional attitudes survey conducted in the UK, compared with men, women were more likely to agree that ‘using animals for food cannot be morally justified’ and were less likely to support the idea that a healthy diet should always include meat^(38)^. Furthermore, when multinomial regression models were run stratified by sex (results not shown), findings suggested that the magnitude and significance of associations for some variables may differ between women and men: for example, high educational attainment predicted meat exclusion among men but not women. Future quantitative and qualitative work is needed to further understand how PBDPs are impacted by intersecting dimensions of identity such as gender, socio-economic status and cultural identity.

The present study also suggests that Canadians living in households where the highest educational attainment level was a bachelor’s degree or higher were more likely to report PBDPs relative to those living in households with lower educational attainment. This finding was not found in other similar studies possibly because those studies measured education of the respondent alone rather than household’s highest education^(9,10)^. However, other studies in America, South Asia, Germany, Italy and Finland with similar methodology to the CCHS reported similar positive associations between higher individual education and vegetarianism^(18,22,23,26,28,34)^. One explanation for the association of vegetarianism with higher educational attainment may be that high education could also indicate high socio-economic status which has been associated with vegetarian status in one Austrian study^(39)^. However, education is only one marker of socio-economic status and other markers such as income and employment status were not associated with PBDPs in the current study. Another explanation could be that better-educated individuals might be more informed regarding the sustainability of meat production which may influence the decision to exclude animal products. One Canadian study which used online convenience sampling to conduct a consumer survey found that those who were more educated were more likely to have changed their opinion on the consumption of beef as environmentally sustainable in the context of increasing beef prices^(40)^. However, the authors mention that reduction of beef consumption was the main focus of their study rather than adherence to diets like veganism or vegetarianism^(40)^.

Compared with all other correlates examined here, self-identified South Asian cultural identity had the strongest association with reporting a PBDP both in the bivariate and multivariable analyses. Vergeer *et al.* found similar results in that participants identifying as South Asian were more likely to report vegetarianism relative to White respondents, Black/Chinese/Aboriginal respondents and respondents who reported ‘Other/mixed’ identities^(10)^. This association may be explained by the relatively high number of PBDP reporters in India (27 % Vegetarian) relative to other countries internationally. The high prevalence of meat-exclusionary dietary practices among South Asians in Canada and India may be due to cultural or religious reasons, with elements of meat exclusion present in Hinduism and Jainism which are practised in India^(41)^. Often vegetarianism in India is a practice one is born into, with similar eating practices passed down through generations^(29)^. Cultural transmission of PBDPs may differ in Western societies where vegetarianism is often framed as a personal dietary choice made for health or ethical reasons^(29)^, rather than a core religious or cultural practice.

Age was not a significant correlate of PBDPs in the current study. This finding was surprising because several studies have suggested associations between vegetarianism and age (though in inconsistent directions). Vegetarians in Germany and Finland tended to be younger, whereas studies from America, South Asia and Italy suggested a correlation between vegetarianism and older age^(18,22,23,26,34)^. One reason for the discrepancies between the present study and others in the literature could be that while a recent Canadian poll has suggested that vegetarians and vegans tend to be younger, it is possible that older adults are also starting to adopt PBDPs as these types of diets have been recommended to mitigate some chronic health conditions that are more prevalent in older populations^(1,7,42)^.

Some study limitations should be considered. The small sample sizes of certain PBDP groups necessitated collapsing of some categorical variables into dichotomous variables to allow for the results to be released by Statistics Canada which resulted in the loss of some precision. An example would be marital status which was collapsed into ‘non-partnered’ and ‘partnered’ categories, wherein the ‘non-partnered’ group included single, widowed and divorced individuals, who may differ in dietary practices. The survey’s response rate was 61·6 % and was accounted for using applied survey weighting in the analysis. This dataset is to our knowledge the best available source of nationally representative PBDP data to date in Canada. Still, there is no way of knowing if the non-responders to the survey differed in rates of adopting PBDPs^(12)^. PBDP measures were also limited, because CCHS never asked respondents about their perceived vegetarian identity, so we cannot compare PBDP definitions based on total exclusion with respondents’ self-perceived adherence to a ‘vegetarian lifestyle’. However, the definitions of PBDP categories were based on exclusion combinations widely accepted in previous vegetarian literature. Future studies aiming to monitor trends in PBDP adherence would benefit from inclusion of questions regarding self-identified PBDP status, the CCHS dietary exclusion question and a question probing temporality of such practices (i.e., how long the respondent has been excluding animal source foods) so the nuances between vegetarian identity, transient PBDP habits and food exclusions can better be explored.

Strengths of the current study include the contribution of the first known prevalence estimates of varied PBDPs in Canada using population representative data from the ten provinces of Canada that can be generalised to the majority of Canadians. It is also the first to create detailed, operationalised definitions of PBDPs using Canadian data that may be replicated in further studies or future iterations of the CCHS. Another strength was the access to and inclusion of many potential correlates drawn from a large dataset, which allowed for broad exploration of the correlates of PBDPs. Finally, it is the first study to explore the demographics of Canadian PBDP followers, facilitating a novel comparison of how the prevalence and correlates of PBDPs in Canada compare to estimates from other countries.

## Conclusion

Despite growing public discourse around PBDPs, only 5 % of Canadians reported PBDPs in 2015. This estimate is far lower than other findings from polling data or studies based on single, poorly defined questions which suggest higher adherence to PBDPs. These findings raise important questions about how prevalence estimates of PBDPs may change depending on sampling approaches, measures and the criteria used to define plant-based diets. Canadians who self-identified as South Asian or belonged to highly educated households were more likely to report pescatarianism, vegetarianism/veganism or red meat exclusion. Using food exclusions, the current study provides a useful approach for constructing and examining several widely described PBDPs that could be applied to future assessment and monitoring of the uptake of dietary practices increasingly being promoted as a strategy to improve healthy and sustainable food consumption.

## Supplementary material

For supplementary material accompanying this paper visit https://doi.org/10.1017/S1368980020003444. click here to view supplementary material
