# Supplementary material for: Plant-based dietary practices in Canada: examining definitions, prevalence and correlates of animal source food exclusions using nationally representative data from the 2015 Canadian Community Health Survey–Nutrition
Source: Public Health Nutr. 2020 Oct 27;24(5):777–86. doi: 10.1017/S1368980020003444 (PMC8025092; doi:10.1017/S1368980020003444)
Supplement: Supplementary file 1 [file S1368980020003444sup001.docx]

**Supplementary Table 1. Weighted Prevalence of Plant-Based Dietary Practices and Reported Exclusions among Canadians aged ≥2 years from the Canadian Community Health Survey-Nutrition 2015 (n= 20,477)**

| **Theoretical Exclusion Category** | **%** | **SE** | **Defined as those who excluded:** |
| --- | --- | --- | --- |
| **Vegan** | 0.28 | 0.08 | Red Meat, Fish, Poultry, Eggs, Dairy |
| **Vegetarian** | 1.29 | 0.16 | Red Meat, Fish, Poultry |
| **Pescatarian** | 0.65 | 0.13 | Red Meat, Poultry |
| **Excluded Red Meat Only** | 2.31 | 0.22 | Red Meat only |
| **Excluded Poultry Only** | 0.20 | 0.06 | Poultry only |
| **Excluded Fish and Shellfish Only** | 5.29 | 0.33 | Fish and Shellfish only |
| **Excluded Eggs Only** | 0.52 | 0.09 | Eggs only |
| **Excluded Dairy Only** | 1.15 | 0.17 | Dairy only |
| **Excluded Gluten Only** | 1.24 | 0.13 | Gluten only |
| **Other Exclusions** | 1.43 | 0.14 | Other combinations of exclusions not captured in the above categories^a^ |
| **Total Exclusions** | 14.30 |  |  |
| **NonExcluders** | 85.63 | 0.47 | Did not report any exclusions |
| **Total** | 99.93^b^ |  |  |
| SE = standard error a) E.g. A respondent who reported excluding only eggs and gluten from their diet would be categorized under “Other exclusions” b) Totals do not equal 100% due to rounding | | | |
